# Editorialets

**Published:** 1908-08

**Authors:** 


### Editorialets.

"Owing to the Inclemency of the Weather."—If any of my readers growl because of the absence of the usual quota of "able editorials" in this issue, let them go and soak their heads. My thermometer broke through the top up at ninety-odd in the shade, and is at the blacksmith's shop getting half soled; that is, I am having another story put on—one story will not register the temperature of Austin. But, all the same, they will find some hot stuff from Lydston in this issue, which must appease their hunger until cooler weather. It is too hot to think. All the other editors are on vacation. It is about as much as I can do to stay here.

---

In renewing his subscription for the twenty-fourth year, Dr. Collins says:

The Grove, Texas, July 21, 1908.

*Dr. F. E. Daniel.*

Inclosed find money order for $1 to renew my subscription. Would be lonesome without the "Red Back."

W. J. Collins.

---

The Square Deal.—Dr. Abbott's Reply to His Critics.—I have received a copy of this virile protest against the outrageous treatment Dr. Abbott has received at the hands of the Octopus. He was singled out for slaughter, and made the recipient of cart loads of vituperation and misrepresentation by the management (editorial) of the monster machine of oppression—the *J. A. M. A.* I had read it previously. In a former issue of the Texas Medical Journal I have expressed my opinion of the editor, the management and the methods of the "Organ,"—denominating it "ye Octopus," a name that will stick to it and irritate its pachyderm like the shirt of Nessus did that of Hercules. Dr. Abbott would do well to reproduce this article, as it was the opening shot of the protest against the medical journal trust. It is leading editorial in my June, 1905, issue. It is a complete expose, and covers the ground. Were I in Dr. Abbott's place I should seek the protection of the courts in a suit, civil and criminal, for libel.

---

"He was a she-goat," as the Dutchman said when inquiring about a strayed sheep. The "Red Back's" staunch friend, Dr. J. T. O'Barr, who has been camping in the mountains, writes, in reply to my inquiry: "The 'big bass that broke my hook and got away' was a catfish, and I caught him, too."

